# Association of DNA repair genes polymorphisms and mutations with increased risk of head and neck cancer: a review

**DOI:** 10.1007/s12032-017-1057-4

**Published:** 2017-11-15

**Authors:** Agata Dylawerska, Wojciech Barczak, Anna Wegner, Wojciech Golusinski, Wiktoria Maria Suchorska

**Affiliations:** 1 Radiobiology Lab, Department of Medical Physics, The Greater Poland Cancer Centre, Garbary 15 Str., 61-866 Poznan, Poland; 2 Department of Head and Neck Surgery, The Greater Poland Cancer Centre, Poznan University of Medical Sciences, Garbary 15 Str., 61-866 Poznan, Poland; 3 Department of Electroradiology, Poznan University of Medical Sciences, Garbary 15 Str., 61-866 Poznan, Poland

**Keywords:** Head and neck cancer, DNA repair, Polymorphisms, Cancer susceptibility

## Abstract

DNA repair mechanisms allow maintain genomic stability and proper functioning within the cells. Any aberrations may cause an increased risk of many diseases such as cancer. The most crucial risk factors for head and neck squamous cell carcinoma are behavioral factors, predominantly chronic exposure to tobacco, alcohol addiction, and infection with human papillomavirus or Epstein–Barr virus. These agents can induce DNA damage; therefore, cells must activate appropriate mechanisms in order to function correctly. Cancer cells are marked with genomic instability, which is associated with a greater tendency for the accumulation of a DNA damage and increased chemo- and radioresistance. Multiple studies have assessed the correlation of increased head and neck cancer (HNC) risk with polymorphism in the DNA repair genes. However, they suggest that interaction of DNA repair genes mutations with susceptibility to HNC depends on a patient’s race and risk factors, especially tobacco smoking. Further identification of these sequence variations must be performed. In this review, we discuss the current knowledge about the DNA repair genes mutations and polymorphisms associated with the high risk of head and neck treatment.

## Introduction

Head and neck cancers (HNC) comprise a group of heterogeneous tumors located in multiple anatomical sites and cellular origins within the head and neck region. Almost 90% of these cancers are squamous cell carcinomas (head and neck squamous cell carcinomas, HNSCC). They include neoplasms of the oral cavity, nasal cavity, pharynx, and larynx. HNC often spread to the lymph nodes of the neck, usually giving the first symptom of the disease [1, 2]. HNSCC remains a major health problem worldwide; it is classified as the sixth most common cancer with around 500,000 new diagnoses each year [3]. In Poland almost 60,000 patients are diagnosed with this disease yearly and nearly 4000 deaths attribute to this type of cancer [4]. The most common symptoms of the HNSCC include: mass in the neck, bleeding from the mouth, sore throat, difficulty food swallowing, cough, and trouble breathing or speaking [5].

A first-line treatment is surgery, as a supportive therapy is frequently used radiation and/or chemotherapy as an adjuvant [6]. HNSCC is strongly associated with certain environmental and lifestyle risk factors such as alcohol consumption, tobacco smoking, UV light, and particular strains of viruses including human papillomavirus (HPV) and Epstein–Barr virus (EBV) [7, 8]. These environmental agents may induce an abnormal DNA damage response (DDR), which can result in cell death, instability of the chromosomes, and unregulated proliferation [9–11]. DNA repair genes play an important role in the maintenance of the genomic integrity and protection of cells from DNA damage. Replication of the damaged DNA contributes to mutation thus causing disease. Therefore, the alteration of DNA repair genes could increase the risk of HNC [12]. The most important pathways include single-strand damage repair and double-strand break (DSB) repair. The aim of this article is to review the polymorphisms and mutations of genes involved in DNA repair systems and role in cancerogenesis of HNC.

## DNA damage and repair: mechanisms for maintaining DNA integrity

**Fig. 1:**
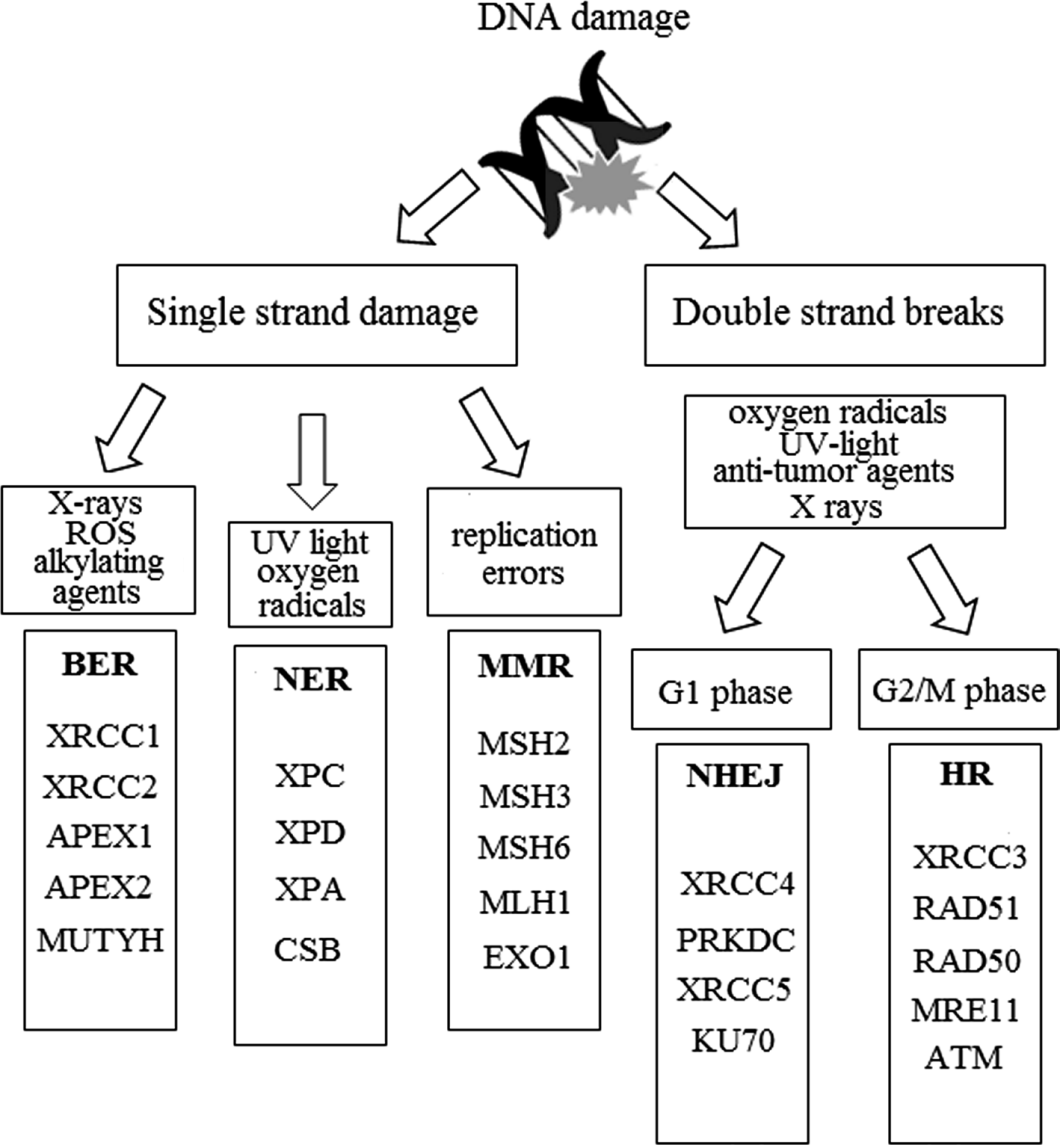
Environmental factors may create DNA damage. The detected lesions are then corrected with the use of two main different mechanisms, depending on the type of damage: single-strand damage repair (SSD) or double-strand breaks system (DSBs). The first one consists of three distinct pathways: BER, NER, and MMR. BER works when DNA alterations include alkylative and oxidative base products, as well as those afforded by radiation. UV light causes damage, which is repaired by NER system, whereas replicative errors are removed by MMR mechanism. DSBs are associated with oxygen radicals, UV light, radiation, and anti-tumor agents. An adequate system is activated depending on the phase of replication process. NHEJ repair is enabled in G1 stage, whereas in G2/M stage HR works

Cells respond to DNA lesions by activation of composite DDR pathways that consist of: cell cycle arrest, transcriptional and posttranslational activation of genes involved in DNA repair and inducing programmed cell death. In case of a great amount of DNA damage accumulated within a cell or incapability to repair all the lesions, there are three possible states, that cell can enter: senescence, apoptosis, or unregulated cell division, which can lead to the formation of a tumor. The efficiency of the DDR pathways’ activation is determined by the nuclear levels of the DNA repair proteins [13, 14]. Incapability of appropriate response to DNA lesions and/or DNA repair leads to genomic instability. The recent studies show that head and neck carcinogenesis is associated with abnormalities in DNA repair, apoptosis, carcinogen metabolism, and cell cycle control [15]. Cells are unable to function properly, if the DNA lesions apply to essential information in the genome. Depending on the type of damage inflicted on the DNA’s double helical structure, a variety of repair strategies have evolved to restore lost information. There are two main pathways of DNA repair: single-strand damage and double-strand breaks (Fig. 1) [13]. Single-strand damage repair is substantially a valid system that requires the presence of an intact DNA strand as a template. It consists of three main types of mechanisms: base excision repair (BER), nucleotide excision repair (NER), and mismatch repair (MMR). BER is activated, when the DNA lesion is localized within the single nitrogenous base. Base modifications are the most common type of endogenous DNA damage, accounting for thousands of lesions per mammalian genome daily. DNA alterations include alkylative and oxidative base products, abasic sites, strand breaks, and misincorporated nucleotides. BER pathway evolved to cope with the high level of spontaneous decay products that are formed in DNA, as well as those damages created upon reactions with natural endogenous substances such as reactive oxygen species (ROS) [16]. This mechanism encompasses following steps: (1) detection and removal of a damaged base by DNA glycosidase, resulting in creation of an apurinic or apyrimidinic site (AP site); (2) cutting the damaged DNA backbone at the AP site by AP endonuclease; (3) removal of the damaged region by lyase or phosphodiesterase; (4) synthesis of the new strand by DNA polymerase using the complementary strand as a template; (5) re-enactment of a phosphodiester bond by DNA ligase [17]. NER leads to removal of a larger DNA fragment comparing to BER. This system repairs DNA lesions, which consist of a helix-distorting damage, such as pyrimidine dimerization caused by UV light. In the first phase proteins XPA (DNA damage recognition and repair factor) and XPC (XPC complex subunit, DNA damage recognition and repair factor) incorporate to the damaged region. Then, two endonucleases—ERCC4 (ERCC excision repair 4, TFIIH core complex helicase subunit) and ERCC5 (ERCC excision repair 5, TFIIH core complex helicase subunit), cut the DNA strand on both sides of the damage. Impaired fragment is then degraded by DNA helicases (ERCC2 (ERCC excision repair 2, TFIIH core complex helicase subunit) and ERCC3 (ERCC excision repair 3, TFIIH core complex helicase subunit)). Finally, as in BER mechanism, the gap is filled with the use of the DNA polymerase (in the case of eukaryotic cells with DNA polymerase δ or ε) and the phosphodiester bond is recreated by DNA ligase [18]. MMR system’s function is to recognize and repair incorrect insertion, deletion, and mis-incorporation of nitrogenous bases that can occur throughout the DNA replication process. The activation of this mechanism depends on the valid functioning of an enzymatic complex that include: MutS protein, which forms a dimer, that recognizes an incorrect complementary between nucleotides and binds to the damaged DNA: MuH, which attaches at hemimethylated sites along the impaired fragment, and MutL protein, which activates the MutH peptide and also acts as a mediator between MutS2 and MutH. At the final step, DNA polymerase and ligase catalyze the synthesis of a new DNA fragment [19]. A crucial part of this system is the MutS-homolog 2 (MSH2) gene. It encodes a protein which recognizes DNA mismatches by forming two functional heterodimers: MSH2–MSH6 and MSH2–MSH3. The first one recognizes single-base mismatches and short insertion–deletion loops, whereas MSH2–MSH3 complex is able to detect larger loops in DNA molecule [20, 21]. Due to the irreversibility of the changes, DSBs are the most adverse and fatal types of damage. Repair of these impairments can be divided into three main steps: detection, signaling, correction. An adequate system is activated depending on the stage of the cell cycle. Non-homologic end joining (NHEJ) mechanism is enabled in G1 phase, whereas in G2/M phase homologic repair (HR) is activated [13]. During the NHEJ pathway, broken ends are bound by Ku70/80 heterodimer, which results in activation of DNA-PKcs. After the ends have been joined together, the XRCC4 (X-ray repair cross-complementing 4)/ligase IV complex completes the final ligation step, and the lesion is repaired [22]. NHEJ system does not require the presence of DNA template and is operative during all the steps of cell cycle [23, 24].

When the HR mechanism works, resecting of the impaired DNA endings by MRE11 (MRE11 homolog, double-strand break repair nuclease) or Exo1 (exonuclease 1) takes place. It leads to formation of a structure called 3′overhang, which is stabilized by RPA (replication protein A) and then loaded on the homologous DSB region by the strand exchange protein RAD51 (RAD51 recombinase) and by BRCA2 (breast cancer 2, DNA repair associated), resulting in the formation of the Holliday junctions intermediate. In mammalian cells, the HR system seems to be less practical compared with NHEJ, but defects in the HR mechanism to enhance cellular radiosensitivity. Since most cells arrest the cell cycle at the G2 phase in response to ionic radiation (IR) exposure and the HR mechanism mainly works in Late-S and G2 phases, inhibiting this would be a good strategy for the new cancer treatment [25].

These processes may function before, during or after the DNA replication. They are able to recognize abnormalities in the DNA structure and to recreate the valid structure of a molecule. A large number of mutations can result in uncontrolled cell division, which contributes to the development of a cancer, apoptosis, increasing the rate of cell aging, or the formation of hereditary disorders [13].

## Role of DNA repair in carcinogenesis and therapy

Unfortunately, the current treatment does not provide sufficient results compared to the resulting side effects, which have a negative impact on patient’s quality of life (QOL). Nowadays, cancer therapeutics can be a major challenge, when it comes to seeking personalized targeted medicine that is both effective and selective to the malignancy [26]. Characteristic feature of a cancer cells is genomic instability which is associated with a greater tendency to accumulation of a DNA damage. Chemotherapy or radiation therapy leads to the DNA lesions of a cancer cells, thus causing their death [27]. However, a part of them is able to survive in genotoxic stress afforded by therapy due to increased DNA repair [28]. They have an impaired DDR pathway so they can only depend on a sole backup pathway for their survival. The use of inhibitors directed against the essential components of this process could be a promising strategy to eradicate cancer cells. Aberrant epigenetic changes in DNA damage repair genes may also serve as therapeutic targets [29, 30]. Numerous studies are currently performed in order to relate the activation of a DNA repair genes in cancer cells to the low effectiveness of therapy.

### Mutations and polymorphisms of DNA repair genes in HNC

DNA repair systems play an important role in protection against carcinogenic agents. Sequence variation in genes regulating these mechanisms may impair their functions and consequently, result in increased cancer susceptibility. Multiple studies have assessed the correlation of HNC increased risk with polymorphism in the DNA repair genes.

### Single-strand damage repair: genes polymorphisms

BER genes play have been considered as a good candidate causing increased susceptibility for HNC, because of their important role in DNA repair; that is, removal of a lesions caused by ROS and other electrophiles, which are most common types of damage [31]. Mahjabeen et al. [32] demonstrated that APEX1 (apurinic/apyrimidinic endodeoxyribonuclease 1) mutations are associated with increased risk of HNC in the Pakistani population. They studied 50 cases of HNSCC among smokers and non-smokers. At the transcript level, three novel mutations (13T>G, Ser129Arg and Val131Gly) of APEX1 were observed. The homozygous and heterozygous genotypes of variants mentioned above seem to be significantly involved in the development of HNC. Moreover, increased expression of APEX1 correlated with tumor size, clinical stage, and positive lymph node metastasis [32]. However, sequence variations in XRCC1 (X-ray repair cross-complementing 1) gene appear to be correlated with HNSCC susceptibility depending on population. Arg194Trp and Arg399Gln polymorphisms were studied in 55 cases of HNSCC in Turkish population, as well as Arg194Trp, Arg280His, and Arg399Gln variations were analyzed in 6719 Chinese patients [33, 34]. In both studies, mutations of XRCC1 gene are not related to HNC development. However, Choudhury’s [35] findings suggest that interaction of tobacco and polymorphisms of XRCC1 and XRCC2 (X-ray repair cross-complementing 2) genes increase the risk of HNSCC. Furthermore, cross talk between these two DNA repair genes might modulate susceptibility toward HNSCC. He investigated the intercommunication of different variants of XRCC1 (Arg399Gln) and XRCC2 (Arg188His) as well as tobacco exposure in the progression of HNSCC in northeast Indian population. The population-based case–control study included 110 HNSCC patients [35]. Other studies analyzed the SNPs XPC A499 V, XPD K751Q, XRCC1 R399Q, and XRCC3 (X-ray repair cross-complementing 3) T241 M among Caucasian population. The aim was to determine if these changes are potential risk factors and indicators of survival. Following results were obtained: (1) XPC A499 V was associated with increased risk of HNSCC, especially laryngeal carcinoma; (2) XPD K751Q was correlated with higher risk of oral SCC among women; (3) wild-type individuals of XRCC3 T241M exhibited an earlier age of onset; (4) XRCC1 R399Q homozygous mutant genotype determined HPV positivity. Moreover, XPD homozygous mutant subjects showed the shortest survival time, which has raised after full-dose radiotherapy. Furthermore, variations of putative risk alleles seemed to act synergistically, raising the risk of HNSCC. Results described above indicate that SNPs of the DNA repair genes (XPC, XPD, XRCC1, and XRCC3) may affect risk and survival of HNSCC [36]. Other studies present similar findings. XPD Asp312Asn and APEX1 Asp148Glu polymorphisms increase the risk for HNC in association with smoking and/or tobacco chewing in a population of northeast India. Das et al. (2015) investigated the effect of the following DNA repair gene variations: XPD Asp312Asn (G>A), APEX1 Asp148Glu (T>G), and MUTYH (mutY DNA glycosylase) Tyr165Cys (G>A). The study included 80 HNC patients and 92 healthy controls. Genotyping with the use of amplification refractory mutation system-PCR (ARMS-PCR) was performed to analyze XPD Asp312Asn (G>A) alteration and PCR using confronting two-pair primers (PCR-CTPP) for APEX1 Asp148Glu (T>G) and MUTYH Tyr165Cys (G>A) mutations. The XPD Asp/Asn genotype increased the risk for HNC by twofold. Interaction between APEX1 Asp/Asp and XPD Asp/Asn as well as MUTYH Tyr/Tyr and XPD Asp/Asn genotypes further increased the risk by 2.9-folds. The risk was further increased in heavy smokers with the XPD Asp/Asn genotype and heavy tobacco chewers with XPD Asn/Asn genotype by 7.7-fold and 10-fold [37].

Abnormalities involving MMR genes occur in microsatellite instability and the accumulation of mutations in proto-oncogenes or tumor suppressor genes. These factors may result in cancer development. Furthermore, it has been observed that decreased expression of MSH2 gene leads to increased frequency of microsatellite instability [38, 39]. Pereira et al. [40] investigated the expression of the MSH2 DNA repair protein in HNSCC collected from 55 cases in Brazil. Patients with locoregional metastatic malignance and lower MSH2 protein levels presented worse survival. It contributes to a higher clinic aggressiveness of HNSCC [40]. Moreover, there is a study showing that inherited MLH1 (mutL homolog 1) c.-93G>A, MSH2 c.211 + 9C>G, MSH3 c.3133G>A, and EXO1 c.1765G>A anomalies of DNA MMR pathway are essential determinants and important patient outcomes’ predictors of HNSCC, notably among smokers [41]. Alternate studies also point that single nucleotide polymorphism (SNP) in hMLH1 promoter is combined with increased risk of tobacco-related oral squamous cell carcinoma (OSCC) in Asian Indians. 242 patients with tobacco-related cancer and 205 healthy controls were genotyped in order to analyze -93 A>G (rs 1800734) variation in hMLH1 promoter. The number of AA genotype was substantially lower in patients comparing to the controls (21.49 vs. 47.8%). In contrast, the frequency of GG genotype was much higher in patients as compared to the healthy controls (41.32 vs. 13.66%). These results indicate that variant G allele is associated with increased risk of OSCC in comparison with the wild-type A allele [42].

### Double-strand breaks: genes polymorphisms

Since DSBs are the most lethal type of DNA damage, even a single one is able to kill a cell or disturb its functioning [43]. DSBs are mostly corrected by either HR or by NHEJ pathway in mammalian cells [44]. Genetic variations of DSBs genes may lead to adverse differences of repair capacity in the general population and healthy individuals [45]. Recently, RAD51C (RAD51 paralog C) gene has been identified as human cancer gene associated with higher risk for inherited breast and ovarian malignancies [46]. It is one of the five analogues of highly conserved RAD51 recombinase which functions as a principal protein in the HR [47, 48]. Scheckenbach et al. [49] analyzed 121 patients with HNSCCs from Germany for germline alterations in RAD51C. All exons and the adjoining introns of this gene were sequenced. Results revealed five distinct heterozygous variations in seven patients (5.8%). One female patient has been identified as a carrier of germline mutation that disrupted the canonical splice acceptor site of exon 5 (c. 706-2A>G). All sequence mutations were single nucleotide modifications. These changes are associated with reduced or absent (c.706-2A>G) function, thus suggesting that germline polymorphisms in genes of the FA pathway could contribute to higher risk of HNSCC development [49].

Kayani et al. [50] found that RAD51 (135G/C, 172 G/T) and XRCC3 (Thr241Met) polymorphisms may be effective biomarkers for genetic susceptibility to HNC. Each variant was genotyped using the polymerase chain reaction-restriction fragment length polymerase (PCR–RFLP) technique. They investigated 200 pathologically confirmed HNC individuals as well as 150 healthy patients. Homozygous variant CC genotype of RAD51 135G/C was associated with a 2.5-fold increased HNC risk, while second variant—RAD51 172G/T, heterozygous genotype was correlated with a 1.68-fold comparing to the controls. The most meaningful result was the Thr241Met alteration of XRCC3 where they observed 16-fold increased HNC risk [50]. However, genetic alterations of the MRN protein complex, which play an important role in DSBs repair and the DNA damage checkpoint activation, do not contribute to a high susceptibility to HNC. RAD50 (RAD50 double-strand break repair protein) and MRE11 genes were analyzed in 358 patients with three different types of HNC from Poland. PCR-SSCP (polymerase chain reaction single-strand conformation polymorphism) method was used to evaluate the coding sequence and exon–intron boundaries of exons 3, 4, 5, 7, 21, and 25 of the RAD50 gene and exons 5, 8, 9, 10, 14, 15, 17, and 19 of the MRE11 gene. Only four SNPs were found within these sequences (see Table 1) [51]. Regrettably, the potential role of the MRE11 gene in human cancers is not well documented. Only Bartkova et al. [52] have described two germline variations: a missense mutation p.R202G and a truncating mutation p.R633X. These studies certify MRE11 gene as a potential candidate associated with high risk of breast cancer in a subset of non-BRCA1/2 families in Denmark [52]. Specific germline polymorphisms predisposing to HNSCC have only been classified in P53 and INK4a/p16 genes. They are concerned with a wide variety of human cancers [53, 54]. More studies must be performed in order to identify HNSCC patients for germline mutations in FA-associated genes. It could provide further insights into “common disease, rare allele” hypothesis [55] and might also apply to HNSCC patients with relatively young age and/or without known risk factors solving the cause of epidemiological phenomenon. Current knowledge about DNA repair genes polymorphisms or mutations is presented in Table 1.

**Table 1:** DNA repair genes polymorphisms/mutations or their products’ sequence in head and neck cancer

Gene	Mechanism	Mutation/polymorphism	High risk factor	Population	References
APEX1	BER	13T>G, Ser129Arg, Val131Gly	Yes	Pakistani	[32]
XRCC1	BER	Arg194Trp, Arg399Gln	No	Turkish	[33]
XRCC1	BER	Arg194Trp, Arg280His, Arg399Gln	No	Chinese	[34]
XRCC1	BER	Arg399Gln	Yes	Indian	[35]
XRCC2	BER	Arg188His	Yes	Indian	[35]
XPC	NER	A499V	Yes	Caucasian	[36]
XPD	NER	K751Q	Yes	Caucasian	[36]
XRCC1	BER	R399Q	Yes	Caucasian	[36]
XRCC3	HR	T241M	Yes	Caucasian	[36]
XPD	NER	Asp312Asn	Yes	Indian	[37]
APEX1	BER	Asp148Glu	Yes	Indian	[37]
MUTYH	BER	Tyr165Cys	Yes	Indian	[37]
MSH2	MMR	No mutation, low expression	Yes	Brazilian	[40]
MLH1	MMR	c.-93G>A	Yes	Brazilian	[41]
MSH2	MMR	c.211 + 9C>G	Yes	Brazilian	[41]
MSH3	MMR	c.3133G>A	Yes	Brazilian	[41]
EXO1	MMR	c.1765G>A	Yes	Brazilian	[41]
hMLH1	MMR	-93 A>G (promoter)	Yes	Asian Indians	[42]
RAD51C	HR	c. 706-2A>G	Yes	German	[49]
RAD50	HR	c.379G>A (ex. 4), c.943G>T (ex. 7), c.3876C>T (ex. 25)	No	Polish	[51]
MRE11	HR	c.1783-86delAG (int. 16)	No	Polish	[51]
MRE11	HR	p.R202G and p.R633X	Yes	Danish	[52]
RAD51	HR	135G/C, 172 G/T	Yes	Pakistani	[50]
XRCC3	HR	Thr241Met	Yes	Pakistani	[50]

## Conclusions

Activation of DNA repair genes in cancer cells is a severe problem. It leads to the invulnerability to therapy thus decreasing efficiency of the treatment. When the DNA’s structure is impaired, cell undergoes a cell cycle arrest. If DNA repair genes are activated and show a high expression, all the lesions can be corrected and the cell can finish a replication process, and if not it is directed to apoptosis pathway or senescence.

The most crucial risk factors for HNSCC are behavioral factors, predominantly chronic exposure to tobacco, alcohol addiction, and infection with HPV or EBV. HNSCC cancer carcinogenesis has been related to anomalies in DNA repair, apoptosis, carcinogen metabolism, and cell cycle control. Heretofore, single-strand repair genes alterations are well documented, when DSBs mechanism still need more accurate studies. However, latest findings suggest that DNA repair genes’ mutations-related susceptibility to HNC depends on a patient’s race. Further identification of these sequence variations and population-specific differences as well as gene–gene interactions may help in high-risk screening of humans exposed to environmental carcinogens and cancer predisposition in different ethnic groups.
